# Current concepts in the diagnosis and management of adolescent idiopathic scoliosis

**DOI:** 10.1007/s00381-020-04608-4

**Published:** 2020-04-21

**Authors:** Daniel Addai, Jacqueline Zarkos, Andrew James Bowey

**Affiliations:** 1 Department of Orthopaedic Spine Surgery, Great North Children’s Hospital, Royal Victoria Infirmary, Newcastle upon Tyne, NE1 4LP England; 2 Newcastle University, Newcastle upon Tyne, UK

**Keywords:** Adolescent idiopathic scoliosis, Current concepts, Spinal fusion, Aetio-pathogenesis

## Abstract

**Background:**

Adolescent Idiopathic Scoliosis (AIS) is a complex 3D structural disorder of the spine that has a significant impact on a person's physical and emotionalstatus. Thus, efforts have been made to identify the cause of the curvature and improve management outcomes.

**Aim:**

This comprehensive review looks at the relevant literature surrounding the possible aetio-pathogenesis of AIS, its clinical features, investigations, surgicalmanagement options, and reported surgical outcomes in anterior spinal fusion, posterior spinal fusion or combined approach in the treatment of AIS.

## Introduction

Adolescent idiopathic scoliosis (AIS) is a complex 3D structural disorder of the spine seen in children from 10 years old until skeletal maturity [1]. According to the Scoliosis Research Society (SRS), AIS is confirmed by a Cobb angle of 10° or more and accompanied by vertebral rotation [2].

Although a method for classifying scoliosis was first described by John Cobb in 1948, further advances in surgical techniques meant that Lawrence Lenke published a new set of guidelines in 2001 allowing surgeons to decide the best method of treatment depending on curve pattern [3]. Whilst recent research largely maintains that AIS is a multifactorial etiological disease, further studies have advanced our understanding of this deformity as multifaceted with a polygenetic background [4]. Surgery for AIS aims to relieve pain and improve function and cosmetics with minimal rates of complications [5].

## Aetio-pathogenesis

AIS is the most prevalent type of scoliosis, with an occurrence rate of 0.47–5.2% [6]. The condition affects 2–4% of adolescents and accounts for approximately 90% of cases of idiopathic scoliosis in adolescents [7]. The prevalence of small curvatures is thought to be equal among girls and boys; however, severe curves are more prevalent in girls [6, 8].

### Genetics

The pathophysiology of AIS is largely unknown; however, several studies suggest a genetic aspect [4]. Studies indicate an increased risk of developing AIS in people who have first degree relatives affected by AIS (prevalence of 6–11%) [9]. Furthermore, twin studies show that monozygotic twins have higher AIS concordance rates (73%) compared with dizygotic twins (36%) [10].

### Oestrogens

Whilst scoliosis at younger ages shows an equal prevalence in males and females, during puberty the sex ratio increases to 8.4/1 (female/male), suggesting a role of sex hormones in the disease [11]. Esposito et al. and Kulis et al. found that blood content of oestradiol was lower in girls with AIS [11, 12]. Furthermore, Mao et al. and Grivas et al. found a tendency of delayed onset of menarche in AIS girls or girls in northern latitudes where AIS rates are higher [13, 14].

### Calmodulin

Several studies show a relationship between elevated platelet calmodulin levels and scoliosis progression [15, 16]. Lowe et al. suggest the platelet changes are related to paraspinous muscle activity and that calmodulin acts as a systemic mediator of contractile tissues [17]. Zhang et al. found that genetic variants of CALM1 gene are associated with AIS susceptibility [18]. However, as there are interactions between calmodulin and melatonin, calmodulin may be involved in the changes in melatonin level and AIS development [19].

### Melatonin

Machida et al. evaluated 90 pinealectomised chickens and found that scoliosis developed in the majority of chickens treated with serotonin, only 6/30 chickens treated with melatonin and in all 30 chickens who had no therapy. Interestingly, they found that the melatonin-treated chickens with scoliosis had less severe curves than those treated with serotonin [20]. In addition, Sadat-Ali et al. found that serum melatonin was significantly lower in AIS patients [21].

### Abnormal skeletal growth and biomechanical theories

AIS initiation and progression rates are the highest among children undergoing their pubertal growth spurt [22]. Yim et al. reported that girls with severe AIS had delayed menarche with faster skeletal growth rates between 12 to 16 years old [23]. Moreover, Kaced et al. found that girls with AIS are generally taller, with a higher weight than healthy controls [24]. Cheung et al. found that after puberty for AIS girls, there was significantly longer corrected height, arm span, and various body segments and significant correlations between anthropometric parameters and curve severity [25].

Asynchronous neuro-osseous growth of the spinal column and cord has also been suggested to play a role in AIS [9]. The observation that the thoracic spine is longer anteriorly than posteriorly in AIS patients, a phenomenon known as relative anterior spinal overgrowth (RASO) or an uncoupled neuro-osseous growth, has now been corroborated with many anatomical and MRI studies [25–30]. Brink et al. evaluated the cause of anterior-posterior length discrepancy and showed that it was a consequence of both anterior and posterior column shortening and whilst the vertebrae contribute to the length discrepancy, it is mostly due to the secondary increased anterior intervertebral discs height [29]. The longitudinal growth of the vertebral bodies in AIS patients is disproportionate and faster than in age-matched controls and mainly occurs by endochondral ossification. On the other hand, the circumferential growth by membranous ossification is slower in both the vertebral bodies and pedicles [31].

The Hueter-Volkmann theory is widely associated with the pathogenesis of scoliosis and suggests that increased pressure on a vertebral epiphyseal growth plate impedes its rate of growth, whereas decreased pressure across the plate accelerates its growth [32]. The theory suggests that on the concave side of the curve, the epiphyseal plates have abnormally high pressures which lead to decreased rates of growth, whereas on the convex side the pressure is less, thus leading to accelerated growth [33]. Stokes et al. later proposed their vicious cycle hypothesis whereby asymmetric loading in a “vicious cycle” causes vertebral wedging during growth in progressive scoliosis curves. Their hypothesis implies that regardless of the initial cause of scoliosis, mechanical factors increase significantly during periods of rapid adolescent growth, when risk of curve progression is greatest [28, 34].

### Low bone mineral density (osteopenia)

Osteopenia in both their axial and peripheral skeleton has been shown to occur in around 30% of AIS patients [35]. Cheng et al. found that areal bone mineral density (aBMD) and volumetric bone mineral density (vBMD) measured at the bilateral lower extremities were significantly lower in AIS patients compared with controls [36]. Additionally, Yip et al. found that osteopenic patients with AIS had significantly higher risk of surgery even after adjustment for menarche status, age and initial Cobb angle [35].

### Vitamin D

As higher levels of vitamin D correlate with greater bone mineral density, several researchers have questioned the role of vitamin D in AIS. Furthermore, recent studies have shown a relationship between gene polymorphisms of vitamin D receptors (VDRs) and low bone mineral density [37]. Suh et al. reported that the VDR *Bsm*I polymorphism is associated with low bone mineral density at the lumbar spine (LSBMD) in girls with AIS [38]. The mean RANKL and RANKL to OPG ratio in AIS patients were also increased compared with control subjects in one study. Furthermore, the RANKL and RANKL to OPG ratios were negatively correlated to the LSBMD and serum OPG levels in both groups and serum OPG levels were positively correlated to the LSBMD in both groups [39]. Balioglu et al. found vitamin D levels were lower in AIS patients and levels were negatively correlated with Cobb’s angle [37]. Moreover, Hampton et al. found 56% of patients had vitamin D levels requiring supplementation [40].

## Clinical features

AIS patients typically present with a deformity of the back, unequal shoulder levels, waistline asymmetry and a rib prominence [41]. Occasionally back pain, not a typical finding in AIS, may be reported [43]. Rightward thoracic curves predominate in the majority of AIS cases; thus, atypical scoliosis curve patterns, combined with rapidly progressing curves or neurological symptoms, should warrant an investigation into a possible underlying lesion [42].

The physical examination includes assessment of curve patterns, shoulder levels and waist asymmetry [1]. Gait and posture are assessed, especially for a short-leg gait due to leg length discrepancy and listing to one side seen in severe curves [41]. The Adams forward bending test may reveal a rib rotational deformity (rib hump) on the convex side of the curve [1]. At this stage, whilst the patient is bending forward, a scoliometer is used to measure the angle of vertebral rotation [43]. An angle of 7° rotatory asymmetry suggests referral for evaluation of scoliosis [1]. As remaining spinal growth is associated with a risk of curve progression in AIS, monitoring growth velocity in every clinical examination is imperative and one of the most reliable methods for this is simple height measurements [44].

## Investigations

Standard radiological images include upright standing posteroanterior (PA) and lateral views [43]. The location of the apex vertebrae should be determined and corresponds to the curves name: cervical, thoracic, thoracolumbar or lumbar curves [43, 45].The main Cobb angle is measured by identifying the largest curve and its two end vertebrae (EV), defined as the maximally tilted vertebrae cephalad and caudal to the curve’s apex [4]. The Cobb method is then utilised by drawing lines along the superior border of the upper EV and the inferior border of the lower EV to form the Cobb angle [4, 43]. Additional imaging, such as magnetic resonance imaging, is reserved for patients with an atypical presentation of AIS suggestive of other underlying aetiologies [43].

The importance of low radiation techniques is paramount in the discussion of AIS as growing spines are subjected to repeated radiation exposure and thus growing concerns of cancer risks. The EOS slot-scanning 2D/3D system, with a 50–80% lower radiation dose compared with conventional radiography, is gaining in popularity with the additional advantage of simultaneous bi-planar imaging allowing 3D reconstruction of the deformity [46, 47].

## Sequelae

The long-term sequelae of untreated AIS are not only physical such as curve progression, back pain and cardiopulmonary problems but also psychosocial issues [10]. It is generally accepted that curves are unlikely to progress in skeletally mature patients with curves less than 30°. However, curves between 30 and 50° have been shown to progress, on average, 10 to 15° over a patient’s lifetime. Moreover, curves over 50° can progress at a rate of 1° per year [48].

## Non-surgical management

Therapy for AIS patients is not only to correct the deformity but also to slow or halt altogether the curve progression. Currently, AIS patients can undergo conservative or surgical management depending on the patient’s skeletal maturity and curve severity. The SRS recommends that AIS patients who have not reached skeletal maturity and have curves less than 25°, or patients who have reached skeletal maturity and have curves less than 45°, be observed through radiological means every 6 months until skeletal mature then every 2 years after that in adulthood [3, 49].

In AIS patients with curves from 25 to 45°, primary therapy may be bracing. The deformity, however, must be flexible in a skeletally immature patient with a Risser stage between 0 and 2 [3]. Although Risser stage and menarche is currently used for the SRS bracing criteria, recent studies have shown that the Sanders Maturity Scale (SMS) is a better predictor of the curve acceleration phase of growth than the Risser stage [50, 51].

The BRAIST study by Weinstein et al. was a multicentre study that compared the outcomes of bracing for at least 18 h a day to observation. They found the rate of treatment success, which was skeletal maturity without curve progression to 50° or more, was 72% after bracing compared with 48% after observation. They also found that longer hours of brace wear showed a positive association with rate of treatment success [52].

The underarm Boston brace (thoraco-lumbo-sacral orthosis) is the most often used brace and it is well tolerated as it can be hidden under clothes. Another option is the Rigo Chêneau orthoses (RCOs) developed with the intent to combine biomechanical forces in three dimensions, including curve derotation [53]. The Milwaukee brace (cervico-thoraco-lumbo-sacral orthosis), on the other hand, is more difficult to hide and less well tolerated and subsequently no longer plays a role in modern AIS bracing techniques [49]. The brace should be applied for 16–20 h a day and success is defined as curve progression less than 5° by the conclusion of treatment [49]. Bracing is continued until the peak growth spurt has stopped indicated by Risser 4 or 2 years after menarche in girls or Risser 5 in boys. After skeletal maturity, curves less than 30° may be discharged as these are not likely to progress. Night-time braces (Charleston and Providence) are worn for 8 to 10 h a night and may be considered for skeletally immature patients with a single major curve of 25 to 35° and an apex below T8 [49].

Label et al. evaluated the in in-brace radiographic correction for patients treated with either the thoraco-lumbo-sacral orthosis (TLSO) or the RCOs. Following bracing, they found that the apical vertebral rotation was significantly reduced by the RCOs when compared with the TLSO by on average 8.2° vs. 4.9° [54]. Another study evaluated the therapeutic success of the RCOs. At treatment onset, patients had an average Cobb angle of 31.97°, Risser score 1.07 and the mean angle of thoracic rotation (ATR) was 10.2°. After an average treatment period of 36 months, the average final Cobb angle was 28.97°, Risser score 4.88 and the ATR was 8.09° [55].

## Surgical management

**Fig. 1:**
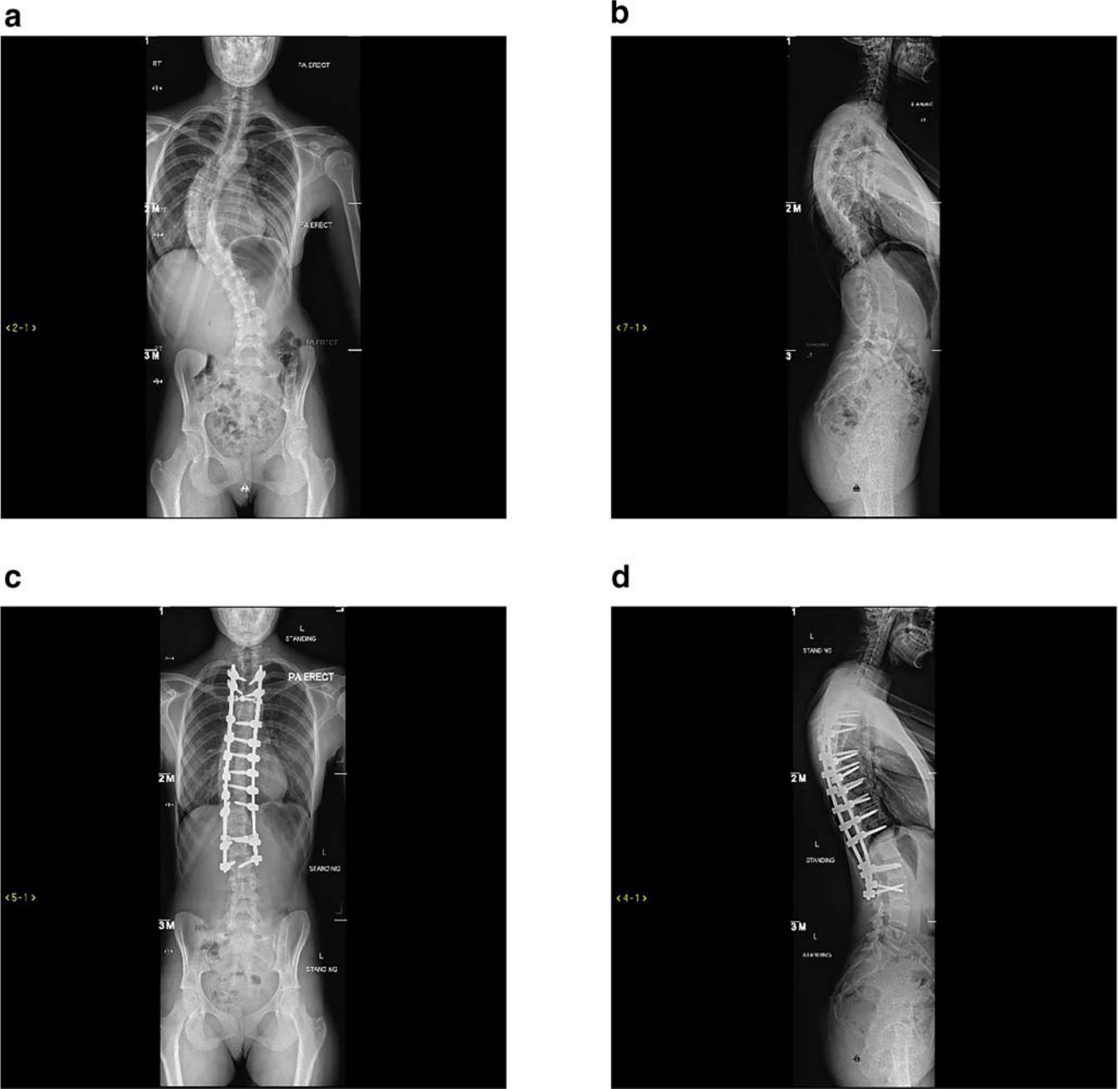
Posterior anterior and lateral preoperative radiographs of a female patient with adolescent idiopathic scoliosis (**a**, **b**) and postoperative radiographs after posterior surgical approach (**c**, **d**)

Whether or not an AIS patient should undergo surgical intervention depends on several factors including the overall curve size and pattern, curve progression and skeletal maturity. Surgery is considered in skeletally immature patients with structural thoracic curve Cobb angles over 40° or patients who show continued progression [56]. For over 100 years, fusion surgery has been used for the treatment of scoliosis [56]. Patients can undergo anterior spinal fusion (ASF), posterior spinal fusion (PSF) (Fig. 1) or a combined anterior/posterior approach. The outcomes and comparisons between these approaches are summarised in Table 1.

**Table 1:** Outcomes of different spinal fusion approaches in the treatment of AIS

Study	Level of evidence	Follow-up time	Approach	Number of patients	Results	Conclusion
Patel et al. [57]	III	Min. 2 years	Anterior	132	• No statistical difference between anterior (48%) and posterior (49%) approaches in SLCC.	Equal SLCC can be reliably achieved with either surgical approach.
			Posterior	44		
Nohara et al. [58]	III	Min. 10 years	Anterior	30	• In PSF, AO occurred in 47%, progression of scoliosis in 7% and disc degeneration in 43%. • In ASF, AO occurred in 53%, progression of scoliosis in 37% and disc degeneration in 53%.	Scoliosis correction was better with ASF immediately postoperatively; however, greater loss of correction occurred at 10 years post op.
			Posterior	30		
Sucato et al. [59]	III	Post op, 1 year and 2 years	Anterior	135	• After surgery, T5–T12 kyphosis was significantly greater after ASF and remained greater at 1 and 2 years post op.	ASF is the best method to restore thoracic kyphosis when compared with PSF.
			Posterior	218		
Tao et al. [60]	III	Post op, 1 year and 2 years	Anterior	21	• Average of 0.61 less segments fused in ASF compared with 0.81 in PSF • SRS-22 scores for pain, self-image/appearance, function/activity, mental and satisfaction of management were significantly higher in ASF group.	ASF results in shorter fusion segments, better sagittal alignment, and QOL than PSF in patients with Lenke type 5 AIS.
			Posterior	26		
Abel et al. [61]	III	2 years	Anterior	40	• PSF had significantly more fused levels than ASF. • ASF had greater percent of lumbar Cobb correction when dLOF was standardised to L3.	Surgeons treating Lenke 5C curves with PSF include more segments. When controlled for the distal level of fixation, ASF provides superior correction of the thoracolumbar curve.
			Posterior	40		
Li et al. [62]	III	Min. 2 years	Anterior	22	• Percent correction of lumbar curve and spontaneous correction of un-fused thoracic curve was similar in both groups. • Fusion levels were significantly shorter in ASF.	There was no statistically significant difference between the 2 approaches in lumbar correction or thoracic correction, but fusion levels were shorter in ASF group.
			Posterior	24		
Miyanjii et al. [63]	II	Min. 2 years	Anterior	69	• No significant differences in percentage correction of the main curve, C7 decompensation, length of hospital stay and SRS outcome scores at 2-year follow-up. • ASF resulted in less levels fused. • PSF resulted in less disc angulation below lowest instrumented vertebrae, greater lumbar lordosis and greater percent correction of lumbar prominence.	The amount of correction achieved was comparable between ASF and PSF. ASF resulted in shorter fusions compared with PSF but there was increased disc angulation below the lowest instrumented vertebrae, less lumbar lordosis, and a lower % correction of the lumbar prominence than PSF.
			Posterior	92		
Rushton et al. [64]	II	2 years	Anterior	18	• No significant differences in the degree of improvement in any areas of the Modified Scoliosis Research Society Outcome Instrument between the groups. • PSF corrected rib hump by 53% and thoracic curve Cobb angle by 62%, whilst ASF corrected rib hump by 61% and thoracic curve Cobb angle by 64%. • The complications were varied and largely intrathoracic in ASF and wound-related in PSF.	Patients with right thoracic AIS of differing curve types but otherwise similar preoperatively demonstrated that ASF and PSF are largely equivalent. Differences in the effect of sagittal alignment, operative time and complications should be considered when selecting approach.
			Posterior	24		
Sudo et al. [65]	III	Average 15.2 years	Anterior	25	• Overall radiographical findings and patient outcome measures were satisfactory. • Average preoperative instrumented level was significantly improved at follow-up; however, average percent-predicted FVC and FEV1 were significantly reduced.	Overall radiographical findings and patient outcome measures of ASF for Lenke 1 MT AIS were satisfactory at an average follow-up of 15 years. Percent-predicted values of FVC and FEV1 were decreased in this cohort, although no patient had complaints related to pulmonary function.
Ghandari et al. [66]	IV	Average 5.6 years	Posterior	42	• Postoperative vertebral tilt below the site of fusion increased from 6.21° ± 5.73° to 11.12° ± 7.92°. • Mean postoperative Oswestry Disability Index (ODI) was 16.7 ± 9.8. • New DDD was observed in 16%.	Despite the efficacy and safety of PSF, it might result in irreversible complications such as DDD. Moreover, the amount of postoperative disability may increase over time.
Luo et al. [67]	III	N/A	Anterior and Posterior approach	308	• No significant differences were noted in correction rate of thoracolumbar/lumbar curve and incidence of proximal junctional kyphosis, in change values of thoracolumbar/lumbar curve and thoracic kyphosis. • ASF had significantly shorter fusion segments. • PSF obtained a larger increasing Cobb angle of lumbar lordosis.	ASF and PSF can obtain similar coronal correction, change values of thoracic kyphosis, and incidence of proximal junctional kyphosis. ASF saves roughly one more fusion segment and PSF can obtain a larger increasing Cobb angle of lumbar lordosis.
Chen et al. [68]	III	N/A	Combined and posterior approach	872	• No significant difference in Cobb angle and percent-predicted FEV1. • Patients in posterior group obtained a better percent-predicted FVC. • Significant less complications, blood loss, operative time and length of hospital stay in posterior group.	Posterior-only approach can achieve similar coronal plane correction and percent-predicted FEV1 compared with combined anterior-posterior approach. Significantly less complication rate, blood loss, operative time, length of hospital stay and better percent-predicted FVC are also achieved by posterior-only approach.
Pourfeizi et al. [69]	III	N/A	Combined	25	• Patients treated through posterior-only and combined approaches were respectively hospitalised for 11.84 ± 5.18 and 26.5 ± 5.2 days • Significant difference between these two groups when considering intensive care unit admission duration, correction in sagittal view of X-ray and number of days the patients underwent traction.	The posterior-only method is associated with some significant advantages and is an advisable method in patients with severe scoliosis over than 70°.
			Posterior	25		
Dobbs et al. [70]	III	Min 2 years	Combined	20	• No statistically significant differences between the number of levels fused, preoperative coronal/sagittal Cobb measurements, coronal curve flexibility or amount of postoperative coronal Cobb correction. • Less of a negative effect on pulmonary function in PSF.	A posterior-only approach has the advantage of providing the same correction as an anterior/posterior spinal fusion, without the need for entering the thorax and more negatively impacting pulmonary function.
			Posterior	34		
Shi et al. [71]	II	3 months, 6 months, 1 year, 2 years and 3 years	Combined	25	• No significant differences in operation time, blood loss, length of hospital stay, SRS-22 Score, coronal curve flexibility or postoperative coronal Cobb correction ratio between approaches. • Implant density was significantly lower in the combined group. • 12 screws were misplaced in the posterior group.	In patients with rigid thoracic AIS, PSF could attain the same curve correction as a combined approach by increasing the implant density. Nevertheless, for patients with a high risk of implant complications, the combined approach is still recommended.
			Posterior	38		

*SLCC*, spontaneous lumbar curve correction; *PSF*, posterior spinal fusion; *AO*, adding-on; *ASF*, anterior spinal fusion; *SRS 30/22*, Scoliosis Research Society Questionnaire; *QOL*, quality of life; *dLOF*, distal level of fixation; *FVC*, forced vital capacity; *FEV1*, forced expiratory volume in 1 s; *DDD*, degenerative disc disease

As can be seen from Table 1, several studies have shown an advantage of the anterior approach in thoracolumbar Lenke 5C curves as it results in less fused levels than the posterior approach. Although there are no reported differences in blood loss, length of hospital stay and patient reported outcomes between both approaches, the posterior approach may save on the negative impacts of the anterior approach on pulmonary function. Studies also showed that the posterior-only approach has the same correction as a combined anterior/posterior spinal fusion, without the need for entering the thorax and thus negatively impacting pulmonary function [57–71].

As fusion limits spinal movement, there is a need for developing motion sparing techniques. A new and promising technique in the surgical management of AIS is vertebral body tethering which utilises patient’s growth to achieve progressive curve correction whilst maintaining patient motion. Samdani et al. evaluated 11 patients with thoracic idiopathic scoliosis and found that average preoperative thoracic Cobb angle of 44.2 ± 9.0° corrected to 20.3 ± 11.0° on first erect with progressive improvement at 2 years [72].

## Conclusions

The aetiology of AIS remains largely unknown; however, several studies show the possible role of genetics, oestrogen, calmodulin, melatonin, vitamin D and low bone mineral density. Furthermore, studies show that AIS progression rates are the highest among those undergoing their pubertal growth spurt, the role of asynchronous neuro-osseous growth in AIS and other biomechanical theories.

The physical examination should include the Adams forward bending test and measurement with a scoliometer and patients with a rotary angle over 7° should be referred to a specialist. Standard radiological imaging and determination of the Cobb angle are used to diagnose and classify the curve as well as evaluate progression. As AIS patients are subjected to frequent radiation exposure, low radiation techniques, such as the EOS system, are gaining in popularity.

The management of AIS includes conservative and surgical options. Bracing shows good outcomes in patients who wear them for a minimum of 18 h a day. In those with curves over 40°, surgery is considered. Though spinal fusion is the traditional approach that is still widely used today, there is promise in vertebral body tethering, a new technique that allows adolescents to maintain their range of motion.

## References

[1] Choudhry M, Ahmad Z, Verma R (2016). Adolescent idiopathic scoliosis. Open Orthop J.

[2] Negrini S, Donzelli S, Aulisa A, Czaprowski D, Schreiber S, de Mauroy J et al. (2018) 2016 SOSORT guidelines: orthopaedic and rehabilitation treatment of idiopathic scoliosis during growth*.* Scoliosis Spinal Disorders 13(1) 10.1186/s13013-017-0145-8 PMC5795289 29435499

[3] Ovadia D (2013). Classification of adolescent idiopathic scoliosis (AIS). J Child Orthop.

[4] Kelly J, Shah N, Freetly T, Dekis J, Hariri O, Walker S et al. (2018) Treatment of adolescent idiopathic scoliosis and evaluation of the adolescent patient. Current Orthopaedic Practice 29(5):424-429

[5] Tambe A, Panikkar S, Millner P, Tsirikos A (2018). Current concepts in the surgical management of adolescent idiopathic scoliosis. Bone Joint J.

[6] Konieczny M, Senyurt H, Krauspe R (2013). Epidemiology of adolescent idiopathic scoliosis. J Child Orthop.

[7] Kikanloo S, Tarpada S, Cho W (2019). Etiology of adolescent idiopathic scoliosis: a literature review. Asian Spine J.

[8] Grauers A, Einarsdottir E, Gerdhem P (2016) Genetics and pathogenesis of idiopathic scoliosis. Scoliosis and Spinal Disorders 11(1) 10.1186/s13013-016-0105-8 PMC5125035 27933320

[9] Cheng JC, Castelein RM, Chu WC, Danielsson AJ, Dobbs MB, Grivas TB, Gurnett CA, Luk KD, Moreau A, Newton PO, Stokes IA (2015). Adolescent idiopathic scoliosis. Nat Rev Dis Prime.

[10] Weinstein S, Dolan L, Cheng J, Danielsson A, Morcuende J (2008). Adolescent idiopathic scoliosis. Lancet.

[11] Esposito T, Uccello R, Caliendo R, Di Martino G, Gironi Carnevale U, Cuomo S (2009). Estrogen receptor polymorphism, estrogen content and idiopathic scoliosis in human: a possible genetic linkage. J Steroid Biochem Mol Biol.

[12] Kulis A, Goździalska A, Drąg J, Jaśkiewicz J, Knapik-Czajka M, Lipik E, Zarzycki D (2015). Participation of sex hormones in multifactorial pathogenesis of adolescent idiopathic scoliosis. Int Orthop.

[13] Mao S, Jiang J, Sun X, Zhao Q, Qian B, Liu Z, Shu H, Qiu Y (2010). Timing of menarche in Chinese girls with and without adolescent idiopathic scoliosis: current results and review of the literature. Eur Spine J.

[14] Grivas T, Vasiliadis E, Mouzakis V, Mihas C, Koufopoulos G (2006) Association between adolescent idiopathic scoliosis prevalence and age at menarche in different geographic latitudes. Scoliosis 1(1) 10.1186/1748-7161-1-9 PMC1501058 16759371

[15] Lowe T, Burwell R, Dangerfield P (2004). Platelet calmodulin levels in adolescent idiopathic scoliosis (AIS): can they predict curve progression and severity?. Eur Spine J.

[16] Kindsfater K, Lowe T, Lawellin D, Weinstein D, Akmakjian J (1994). Levels of platelet calmodulin for the prediction of progression and severity of adolescent idiopathic scoliosis. J Bone Joint Surg.

[17] Lowe T, Lawellin D, Smith D, Price C, Haher T, Merola A, O'Brien M (2002). Platelet calmodulin levels in adolescent idiopathic scoliosis. Spine.

[18] Zhang Y, Gu Z, Qiu G (2014). The association study of calmodulin 1 gene polymorphisms with susceptibility to adolescent idiopathic scoliosis. Biomed Res Int.

[19] Cheung K, Wang T, Qiu G, Luk K (2008). Recent advances in the aetiology of adolescent idiopathic scoliosis. Int Orthop.

[20] Machida M, Dubousset J, Imamura Y, Iwaya T, Yamada T, Kimura J (1995). Role of melatonin deficiency in the development of scoliosis in pinealectomised chickens. J Bone Joint Surg Brit.

[21] Sadat-Ali M, Al-Habdan I, Al-Othman A (2000). Adolescent idiopathic scoliosis. Is low melatonin a cause?. Joint, Bone, Spine: Revue Rhumatisme.

[22] Schlösser T, Vincken K, Rogers K, Castelein R, Shah S (2014). Natural sagittal spino-pelvic alignment in boys and girls before, at and after the adolescent growth spurt. Eur Spine J.

[23] Yim A, Yeung H, Hung V, Lee K, Lam T, Ng B, Qiu Y, Cheng JC (2012). Abnormal skeletal growth patterns in adolescent idiopathic scoliosis—a longitudinal study until skeletal maturity. Spine..

[24] Kaced H, Hanene B, Haddouche A (2017). Abnormal skeletal growth patterns in adolescent idiopathic scoliosis. Med Technol J.

[25] Cheung C, Lee W, Tse Y, Tang S, Lee K, Guo X (2003). Abnormal peri-pubertal anthropometric measurements and growth pattern in adolescent idiopathic scoliosis: a study of 598 patients. Spine..

[26] Somerville E (1952). Rotational lordosis: the development of the single curve. J Bone Joint Surg British.

[27] Roaf R (1966). The basic anatomy of scoliosis. J Bone Joint Surg British.

[28] Fadzan M, Bettany-Saltikov J (2017). Etiological theories of adolescent idiopathic scoliosis: past and present. Open Orthop J.

[29] Brink R, Schlösser T, van Stralen M, Vincken K, Kruyt M, Hui S, Viergever MA, Chu WCW, Cheng JCY, Castelein RM (2018). Anterior-posterior length discrepancy of the spinal column in adolescent idiopathic scoliosis—a 3D CT study. Spine J.

[30] Lao L, Shen J, Chen Z, Wang Y, Wen X, Qiu G (2011). Uncoupled neuro-osseous growth in adolescent idiopathic scoliosis? A preliminary study of 90 adolescents with whole-spine three-dimensional magnetic resonance imaging. Eur Spine J.

[31] Guo X, Chau W, Chan Y, Cheng J (2003). Relative anterior spinal overgrowth in adolescent idiopathic scoliosis. J Bone Joint Surg British.

[32] Stokes I, Mente PL, Iatridis JC, Farnum CE, Aronsson DD (2002). Enlargement of growth plate chondrocytes modulated by sustained mechanical loading. JBJS..

[33] Stokes I (2007). Analysis and simulation of progressive adolescent scoliosis by biomechanical growth modulation. Eur Spine J.

[34] Stokes I, Burwell R, Dangerfield P (2006) Biomechanical spinal growth modulation and progressive adolescent scoliosis–a test of the ‘vicious cycle’ pathogenetic hypothesis: summary of an electronic focus group debate of the IBSE. Scoliosis 1(16) 10.1186/1748-7161-1-16 PMC1626075 17049077

[35] Yip B, Yu F, Wang Z, Hung V, Lam T, Ng B et al. (2016) Prognostic Value of Bone Mineral Density on Curve Progression: A Longitudinal Cohort Study of 513 Girls with Adolescent Idiopathic Scoliosis. Scientific Reports. 19 (6) 10.1038/srep39220 PMC5171643 27991528

[36] Cheng J, Qin L, Cheung C, Sher A, Lee K, Ng S, Guo X (2000). Generalized low areal and volumetric bone mineral density in adolescent idiopathic scoliosis. J Bone Miner Res.

[37] Balioglu M, Aydin C, Kargin D, Albayrak A, Atici Y, Tas S (2016). Vitamin-D measurement in patients with adolescent idiopathic scoliosis. J Pediatr Orthop B.

[38] Suh K, Eun I, Lee J (2010). Polymorphism in vitamin D receptor is associated with bone mineral density in patients with adolescent idiopathic scoliosis. Eur Spine J.

[39] Suh K, Lee S, Hwang S, Kim S, Lee J (2007). Elevated soluble receptor activator of nuclear factor-κB ligand and reduced bone mineral density in patients with adolescent idiopathic scoliosis. Eur Spine J.

[40] Hampton M, Evans O, Armstrong S, Naylor B, Breakwell L, Cole A (2016). Prevalence and significance of vitamin D deficiency in patients with adolescent idiopathic scoliosis requiring corrective surgery. Spine J.

[41] Altaf F, Gibson A, Dannawi Z, Noordeen H (2013). Adolescent idiopathic scoliosis. Br Med J.

[42] Kim W, Porrino J, Hood K, Chadaz T, Klauser A, Taljanovic M (2019). Clinical evaluation, imaging, and management of adolescent idiopathic and adult degenerative scoliosis. Curr Probl Diagn Radiol.

[43] Burton M (2013). Diagnosis and treatment of adolescent idiopathic scoliosis. Pediatr Ann.

[44] Janicki J, Alman B (2007). Scoliosis: review of diagnosis and treatment. Paediatr Child Health.

[45] Greiner KA (2002). Adolescent idiopathic scoliosis: radiologic decision-making. Am Fam Physician.

[46] Lau L, Hung A, Chau W, Hu Z, Kumar A, Lam T, Chu WCW, Cheng JCY (2019). Sequential spine-hand radiography for assessing skeletal maturity with low radiation EOS imaging system for bracing treatment recommendation in adolescent idiopathic scoliosis: a feasibility and validity study. J Child Orthop.

[47] Bagheri A, Liu X, Tassone C, Thometz J, Tarima S (2018). Reliability of three-dimensional spinal modeling of patients with idiopathic scoliosis using EOS system. Spine Deformity.

[48] Reamy BV, Slakey JB (2001) Adolescent idiopathic scoliosis: review and current concepts. American Family Physician 64(1):111-116 11456428

[49] Zheng Shuhui, Zhou Hang, Gao Bo, Li Yongyong, Liao Zhiheng, Zhou Taifeng, Lian Chengjie, Wu Zizhao, Su Deying, Wang Tingting, Su Peiqiang, Xu Caixia (2018). Estrogen promotes the onset and development of idiopathic scoliosis via disproportionate endochondral ossification of the anterior and posterior column in a bipedal rat model. Experimental & Molecular Medicine.

[50] Neal K, Shirley E, Kiebzak G (2018). Maturity indicators and adolescent idiopathic scoliosis. SPINE..

[51] Minkara A, Bainton N, Tanaka M, Kung J, DeAllie C, Khaleel A et al (2018) High risk of mismatch between Sanders and Risser staging in adolescent idiopathic scoliosis. J Pediatr Orthop 1 10.1097/BPO.0000000000001135 31923164

[52] Weinstein S, Dolan L, Wright J, Dobbs M (2013). Effects of bracing in adolescents with idiopathic scoliosis. N Engl J Med.

[53] Minsk M, Venuti K, Daumit G, Sponseller P (2017) Effectiveness of the Rigo Chêneau versus Boston-style orthoses for adolescent idiopathic scoliosis: a retrospective study. Scolio Spinal Disorders 12(1) 10.1186/s13013-017-0117-z PMC5357818 28331904

[54] Lebel D, Al-Aubaidi Z, Shin E, Howard A, Zeller R (2013). Three dimensional analysis of brace biomechanical efficacy for patients with AIS. Eur Spine J.

[55] Ovadia D, Eylon S, Mashiah A, Wientroub S, Lebel E (2012). Factors associated with the success of the Rigo System Chêneau brace in treating mild to moderate adolescent idiopathic scoliosis. J Child Orthop.

[56] Wagner S, Lehman R, Lenke L (2015). Surgical management of adolescent idiopathic scoliosis. Seminars Spine Surg.

[57] Patel P, Upasani V, Bastrom T, Marks M, Pawelek J, Betz R, Lenke LG, Newton PO (2008). Spontaneous lumbar curve correction in selective thoracic fusions of idiopathic scoliosis. Spine..

[58] Nohara A, Kawakami N, Saito T, Tsuji T, Ohara T, Suzuki Y, Tauchi R, Kawakami K (2015). Comparison of surgical outcomes between anterior fusion and posterior fusion in patients with AIS Lenke type 1 or 2 that underwent selective thoracic fusion -long-term follow-up study longer than 10 postoperative years. Spine..

[59] Sucato D, Agrawal S, O’Brien M, Lowe T, Richards S, Lenke L (2008). Restoration of thoracic kyphosis after operative treatment of adolescent idiopathic scoliosis. Spine..

[60] Tao F, Wang Z, Li M, Pan F, Shi Z, Zhang Y, Wu Y, Xie Y (2012). A comparison of anterior and posterior instrumentation for restoring and retaining sagittal balance in patients with idiopathic adolescent scoliosis. J Spinal Disord Tech.

[61] Abel M, Singla A, Feger M, Sauer L, Novicoff W (2016). Surgical treatment of Lenke 5 adolescent idiopathic scoliosis: comparison of anterior vs posterior approach. World J Orthop.

[62] Li M, Ni J, Fang X, Liu H, Zhu X, He S (2009). Comparison of selective anterior versus posterior screw instrumentation in Lenke5C adolescent idiopathic scoliosis. Spine..

[63] Miyanji F, Nasto L, Bastrom T, Samdani A, Yaszay B, Clements D, Shah SA, Lonner B, Betz RR, Shufflebarger HL, Newton PO (2018). A detailed comparative analysis of anterior versus posterior approach to Lenke 5C curves. Spine..

[64] Rushton P, Grevitt M, Sell P (2015). Anterior or posterior surgery for right thoracic adolescent idiopathic scoliosis (AIS)? A prospective cohorts’ comparison using radiologic and functional outcomes. J Spinal Disord Tech.

[65] Sudo H, Ito M, Kaneda K, Shono Y, Takahata M, Abumi K (2013). Long-term outcomes of anterior spinal fusion for treating thoracic adolescent idiopathic scoliosis curves. Spine..

[66] Ghandhari H, Ameri E, Nikouei F, Haji Agha Bozorgi M, Majdi S, Salehpour M (2018) Long-term outcome of posterior spinal fusion for the correction of adolescent idiopathic scoliosis. Scoliosis and Spinal Disorders 13(1) 10.1186/s13013-018-0157-z PMC6090875 30123840

[67] Luo M, Wang W, Shen M, Xia L (2016) Anterior versus posterior approach in Lenke 5C adolescent idiopathic scoliosis: a meta-analysis of fusion segments and radiological outcomes. J Orthop Surg Res 11(1) 10.1186/s13018-016-0415-9 PMC4940871 27401875

[68] Chen Z, Rong L (2016). Comparison of combined anterior–posterior approach versus posterior-only approach in treating adolescent idiopathic scoliosis: a meta-analysis. Eur Spine J.

[69] Pourfeizi H, Sales J, Tabrizi A, Borran G, Alavi S (2014). Comparison of the combined anterior-posterior approach versus posterior-only approach in scoliosis treatment. Asian Spine J.

[70] Dobbs M, Lenke L, Kim Y, Luhmann S, Bridwell K (2006). Anterior/posterior spinal instrumentation versus posterior instrumentation alone for the treatment of adolescent idiopathic scoliotic curves more than 90°. Spine..

[71] Shi Z, Chen J, Wang C, Li M, Li Q, Zhang Y, Li C, Qiao Y, Kaijin G, Xiangyang C, Ran B (2015). Comparison of thoracoscopic anterior release combined with posterior spinal fusion versus posterior-only approach with an all-pedicle screw construct in the treatment of rigid thoracic adolescent idiopathic scoliosis. J Spinal Disord Tech.

[72] Samdani A, Ames R, Kimball J, Pahys J, Grewal H, Pelletier G, Betz RR (2014). Anterior vertebral body tethering for idiopathic scoliosis. Spine..

